# Balloon Dilatation of Pediatric Subglottic Laryngeal Stenosis during the Artificial Apneic Pause: Experience in 5 Children

**DOI:** 10.1155/2014/397295

**Published:** 2014-06-12

**Authors:** J. Lisý, D. Groh, M. Chovanec, M. Marková, V. Suchánek, P. Polášková, M. Trávníček

**Affiliations:** ^1^Department of Imaging Methods, 2nd Medical Faculty, University Hospital Motol, Charles University, 150 06 Prague, Czech Republic; ^2^Children ENT Department, 2nd Medical Faculty, University Hospital Motol, Charles University, 150 06 Prague, Czech Republic; ^3^Department of Otorhinolaryngology and Head and Neck Surgery, 1st Medical Faculty, University Hospital Motol, Charles University, 150 06 Prague, Czech Republic; ^4^Department of Anesthesiology, 2nd Medical Faculty, University Hospital Motol, Charles University, 150 06 Prague, Czech Republic

## Abstract

*Introduction*. Balloon dilatation is a method of choice for treatment of laryngeal stenosis in children. The aim of procedure in apneic pause is to avoid new insertion of tracheostomy cannula. *Patients and Methods*. The authors performed balloon dilatation of subglottic laryngeal strictures (SGS) in 5 children (3 girls and 2 boys) without tracheotomy. Two of them with traumatic and inflammatory SGS had a tracheal cannula removed in the past. The other 3 children with postintubation SGS had never had a tracheostomy before. The need for tracheostomy due to worsening stridor was imminent for all of them. *Results*. The total of seven laryngeal dilatations by balloon esophagoplasty catheter in apneic pause was performed in the 5 children. The procedure averted the need for tracheostomy placement in 4 of them (80%). Failure of dilatation in girl with traumatic stenosis and concomitant severe obstructive lung disease led to repeated tracheostomy. *Conclusion*. Balloon dilatation of laryngeal stricture could be done in the absence of tracheostomy in apneic pause. Dilatation averted threatening tracheostomy in all except one case. Early complication after the procedure seems to be a negative prognostic factor for the outcome of balloon dilatation.

## 1. Introduction

Balloon dilatation is a method of choice in the treatment of laryngeal stenosis in children. When tracheostomy is present, dilatation is performed under general anesthesia; in this case, ventilation is ensured through the tracheostomy. The aim of the dilatation procedure is to allow subsequent removal of the tracheostomy.

Balloon dilatation during apneic pause is beneficial to children who had never had a tracheostomy as well as to children who had a tracheostomy previously removed and are experiencing worsening of clinical symptoms. In the former, balloon dilatation presents a noninvasive treatment option. In the latter, it can prevent the need for reinsertion.

## 2. Material and Methods

Balloon dilatation was used for treatment of five children with subglottic laryngeal stenosis (SGS) who suffered from worsening of dyspnea with stridor and would otherwise be indicated for tracheotomy or other optional surgical treatments. Two patients had a tracheostomy cannula previously removed followed by a plastic surgery of the tracheostomy site (one boy with inflammatory stenosis and one girl with traumatic stenosis). The other three children had never had a tracheostomy cannula (two girls and one boy with subglottic stricture after long lasting intubation).

Balloon dilatation of laryngeal stricture is a modification of a method used for esophageal strictures (3). Laryngeal strictures are shorter; therefore, shorter balloon can be used. The catheter is guided under laryngoscopic control, sometimes with a help of Magill forceps. General anesthesia with deeper relaxation compared to esophageal dilatations is beneficial. Angiographic guide wire reinforces the catheter but is not used for the placement of balloon into the stricture. Ventilation during the procedure is usually possible through tracheostomy cannula which is applied to the majority of patients.

Balloon catheters with the balloon diameter ranging from 10 to 23 mm (Accent, Cook), identical to the ones used for esophageal dilation, were used. The catheter was introduced transorally under laryngoscopic control, eventually with the help of Magill's forceps.

The balloon was positioned into the center of the stenosis and patients were hyperventilated with oxygen delivered by assisted mask ventilation for one minute. During the following apneic pause the balloon was distended by water soluble nonionic contrast media. The apneic pause lasted no more than 45 seconds or until the patient's SpO2 dropped to 90%, at which point the airway was reassessed.

Later after the deflation of the balloon the patient was again hyperventilated for 1 minute. Balloon inflation was repeated six times. The time of laryngeal occlusion must be closely monitored in children without tracheotomy. Patients were monitored by pulse oximetry and ECG during the procedure. If the patient breathes and eats comfortably in the recovery unit, next day discharge home can be safely achieved. We did not use mitomycin C or local steroids in the management of SGS, as there are no prospective randomized studies demonstrating their benefit and there are concerns of potential local noxious effects.

The primary positive outcome was achieving a functional airway without the need of an open laryngotracheal surgery or ongoing need for a tracheostomy. The negative outcome was defined as the need for a new tracheostomy or optional open surgery.

## 3. Results

The total of seven laryngeal dilatations by standard balloon esophagoplasty catheter in apneic pause was performed in the 5 children (repeated dilatation was necessary in one boy with inflammatory SGS and girl with postintubation SGS). The procedure averted the need for tracheostomy placement in 4 of them (80%) (Table 1). None of the patients suffered a major complication related to balloon dilatation. Failure of dilatation in girl with traumatic stenosis and concomitant severe obstructive lung disease led to repeated tracheostomy.

### 3.1. Case 1

One-year-old girl suffered previously a trauma in a car accident. The traumatic impairment of bilateral laryngeal innervations resulted in development of SGS requiring tracheostomy placement. Repeated vaporizations by laser including lateral fixation of left ventricular plica allowed the removal of the cannula. However, immobility of glottis with laryngeal stricture resulted in breathing with a stridor.

**Figure 1:**
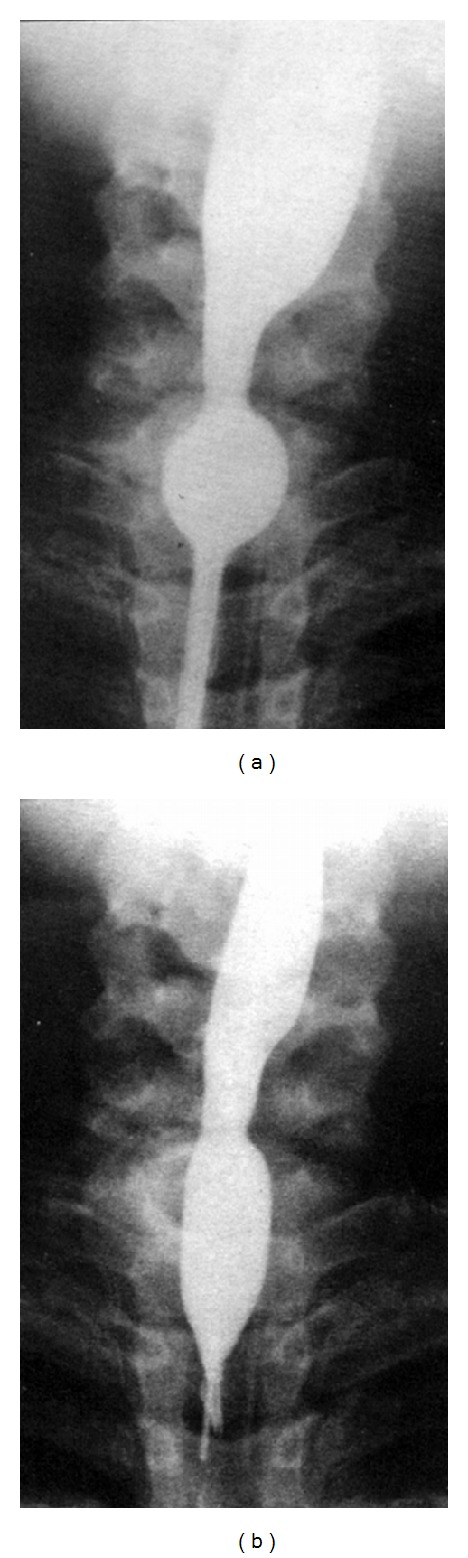
(a) Balloon dilation in apneic pause was performed using 20 mm balloon with mild effect. (b) Replacement of catheters by another with 15 mm balloon allowed achieving more promising result.

Balloon dilatation in apneic pause was performed using 20 mm balloon with mild effect (Figure 1(a)). Replacement of the catheter by another with 15 mm balloon led to more promising result (Figure 1(b)).

The girl experienced respiratory insufficiency one hour after dilatation which required artificial ventilation for 1 day. Spirometry showed severe obstructive lung disease. The tracheostomy cannula had to be reinserted again 40 days after balloon dilatation.

### 3.2. Case 2

Girl, 13 years old, had a history of SGS lasting 10 years after repeated intubations following a cardiac surgery. She never had a tracheostomy cannula before. Worsening of dyspnea with stridor led to dilatation in the apneic pause by 18 mm balloon, followed by a second dilatation using 23 mm balloon. The girl has been symptoms-free since the second procedure.

## 4. Discussion

The incidence of subglottic laryngeal stenosis (SGS) has decreased in time to 1-2% in 2000 due to advances in airway management and guidelines for intubation [1]. However, the management of SGS in children continues to be a challenging problem for the otolaryngologist. Management options for SGS vary from observation to surgical intervention, with the goals being to either bypass the stenotic segment by tracheotomy or increase the diameter of the subglottic airway by cricoid split, laryngotracheal reconstruction, or partial cricotracheal resection. In the last years, balloon dilatation which offers the benefit of reduced invasiveness became an alternative to open laryngeal procedures. However, reported success rates are variable. The first description of balloon catheter technology used for tracheal stenosis appeared in 1984 [2]. Balloon tracheoplasty for treatment of subglottic laryngeal stenosis was reported in 1991 by Hebra et al. [3].

Balloon laryngoplasty was proved as an effective, stand-alone procedure for the management of SGS in 70% of patients, obviating the need for tracheotomy or cricoid split [4]. Others showed 65% successful outcome of primary balloon dilatation of SGS, higher in acquired (70%) than in congenital (50%) stenosis [5]. Another study of primary balloon dilatation in the endoscopic management of pediatric SGS showed that in 60% of cases it was possible to avoid an open reconstruction or tracheotomy and 40% of patients had their symptoms temporarily improved until definitive open reconstruction. [6]. Success rate of balloon dilatation is decreasing in time as was shown by Bent et al., with stridor eliminated or greatly improved in all patients the first day after the procedure, which was lasting in 70% of patients during follow-up [7].

Large systematic review of the literature evaluated 22 relevant studies on balloon and rigid dilatation as primary therapy for laryngotracheal stenosis (LTS) in pediatric patients [8]. Although it showed that balloon dilatation alone is less successful (success rate 50%) than balloon dilatation with adjuvant therapy (the Carbon dioxide or KTP laser, topical or intralesional injected steroid; success rate ranged from 50% to 78%), success rate of balloon dilatation alone in our patients was 80%.

We have found only one case report about a balloon dilatation of subglottic stenosis with noninvasive ventilation [9]. Concomitant airway disorder was shown as a negative prognostic factor when 75% of failed balloon dilatations were in children who had concomitant airway disorders; in contrast, only 37.5% of successfully treated children had concomitant airway disorders (*P* = 0.048) [6]. This observation helps to understand the failure of balloon dilatation in one-year-old girl with traumatic subglottic laryngeal stenosis after a car accident who suffered from severe obstructive lung disease.

## 5. Conclusion

Balloon dilatation of a subglottic laryngeal stenosis can be safely performed in those patients where the worsening of dyspnea and stridor would lead to tracheostomy cannula insertion. Balloon dilatation of laryngeal stenosis in apneic pause allowed achieving sufficient diameter of laryngeal lumen in 80% of children with subglottic stenosis and avoiding threatening tracheostomy placement or alternative surgical method without any significant complication after the procedure. Balloon dilatation was unsuccessful in a girl with traumatic subglottic stenosis and concomitant severe obstructive lung disease. It seems that an occurrence of a complication early after the procedure is a negative prognostic factor of the outcome of treatment.

## Figures and Tables

**Table 1:** 

Age (years)	1	8	9	11	13

Gender	F	F	M	M	F

Etiology	Trauma	Intubation	Intubation	Inflammation	Intubation

Previous tracheostomy	Yes	No	No	Yes	No

Dilatations (number)	1	1	1	2	2

Balloon width (mm)	20 and 15 (∗)	15 and 10 (∗)	18	18 and 18	18 and 23

Width of stenosis (mm)	5 and 8 (∗)	7 and 9 (∗)	9	8 and 10	10 and 12

Complications	Respiratory insufficiency	—	—	—	—

Follow-up (years)	Retracheostomy	12	2	4	3

(∗) Two balloons used during one dilatation.
